# From Genomes to GENE-omes: Exome Sequencing Concept and Applications in Crop Improvement

**DOI:** 10.3389/fpls.2017.02164

**Published:** 2017-12-19

**Authors:** Parampreet Kaur, Kishor Gaikwad

**Affiliations:** National Research Centre on Plant Biotechnology, New Delhi, India

**Keywords:** exome, whole genome sequencing, whole exome sequencing, crop improvement, genomics

## Abstract

Exome sequencing represents targeted capture and sequencing of 1–2% of ‘high-value genomic regions’ (subset of the genome) which are enriched for functional variants and harbors low level of repetitive regions. We discuss here an overview of exome sequencing, ways to approach plant exomes, and advantages and applicability of this powerful approach in deciphering functional regions of genomes. Though initially this approach was developed as an alternative to whole genome sequencing (WGS), but the multitude of benefits conferred by sequence capture via hybridization approaches created a niche for itself to solve many of biological riddles, particularly for resolving phylogenetic distances. The technique has also proved to be successful in understanding the basis of natural and induced molecular variation, marker development and developing genomic resources for complex, wild and non-model species, which are still intractable for WGS efforts. Thus, with profound applications of this powerful sequencing strategy, near future is expected to witness a collective expansion of both techniques, i.e., sequence capture via hybridization for evolutionary and ecological research and WGS approaches for its universal accessibility.

## Introduction

Completion of an Arabidopsis plant genome sequence in 2000 marked a major breakthrough in the sequencing era. Further progress in deciphering of large and complex genomes of crop species is a reflection of the advancement of sequencing technologies that are commonly referred to as NextGen sequencers along with the evolution of more powerful data analysis tools.

Polyploidization has played a critical role during evolution in shaping the complex genomes of important crop species viz. Wheat, Brassica etc., and has resulted in large genome sizes with exceptionally high repeat content, heterozygosity and presence of closely related homeologous genes in large gene families. This poses significant challenge for whole genome sequencing (WGS) and resequencing in many such plant lineages. Several strategies such as reduction of genome complexity, sequencing of diploid progenitors and sequencing of sorted and purified chromosome arms are thus deployed to gain chromosome specific insight into genomes of complex polyploids (Bevan et al., 2017). One important way to achieve reduction in genome complexity analysis is referred to as Sequence Capture or Targeted Sequencing that includes either specific genes of interest or targets within genes or the entire protein coding region of genome, i.e., exome and could be achieved using either of the three modes: hybridization based sequence capture, PCR-based amplification, and selective circularization (Dahl et al., 2005; Hodges et al., 2007; Gnirke et al., 2009). Among these, hybrid capture mode allows several megabases of DNA to be analyzed for efficient sequencing of the complete ‘exome’ and represents its current major application. Additionally, the technique has advantage of being extraordinarily quick, simple, inexpensive, and requires small amount of input DNA (<1–3 μg) (Mertes et al., 2011).

Exome sequencing restricts attention only to the genomic fraction that encodes for mRNA and eventually a phenotype, and is thus considered to be adequate to explain the molecular origin of genetic variation (induced/natural). Coding sequences represents only 1–2% of genome depending on species (Warr et al., 2015) and comprises high level of functional variants and low repeat content. For example, wheat exome constitute 170–340 Mb (Paux et al., 2006) of size in comparison to massively large genomes of tetraploid (10 Gb) and hexaploid (17 Gb) wheat. Considering the manageable size of many crop exomes and by emphasizing on ‘high-value genomic regions’ (Hodges et al., 2007), exome analysis permits deep sequencing for large number of samples to identify useful variants for incorporation in molecular breeding strategies.

## How to Approach Plant Exome

Two technological alternatives available for hybridization based exome capture are: array/chip- based capture (Hodges et al., 2007; Okou et al., 2007) and In solution capture (Gnirke et al., 2009). Both approaches relies on specifically designed probes or baits for target enrichment from sequencing library, but solution captures requires more concentration of baits over DNA library in contrast to array capture that requires excess of library over probes to perform enrichment.

**FIGURE 1:**
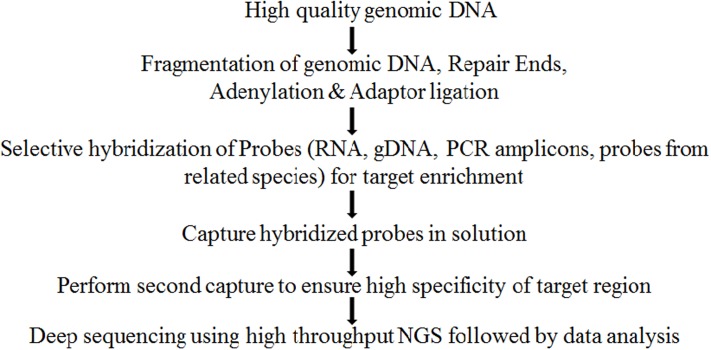
A general outline for exome sequencing.

Exome sequencing could be divided into two phases, first phase constitutes probe hybridization for selection of subset of DNA that encodes for a protein, i.e., target enrichment and second phase constitutes its high throughput DNA sequencing (**Figure 1**). Certain factors such as mode and quality of processing of input DNA sample, number of targets, coverage depth for each target, probe design and GC content (Zhou and Holliday, 2012), expected enrichment efficiency, sequencing technology used and biological system studied etc., should be considered while designing an exome capture experiment (Mertes et al., 2011; Grover et al., 2012). Of all these factors, depth of the sequence coverage is important in achieving good reliability of sequencing of exome capture experiments and requires coverage of atleast 30X or higher to successfully validate the variation identified (Winfield et al., 2012). Coverage depth in turn is governed by probe specificity, homeology and heterozygosity level, ploidy level, genome size, presence of orthologs and paralogs as well as by characteristics of probes such as genomic regions from where the probe is designed, i.e., whether conserved or unique (Grover et al., 2012). Well-established strategies are available for construction of exome libraries along with ready to use exome kits (NimbleGen, Affymetrix) and their user dependent customizations are available for many crop species such as soybean, wheat, barley, and maize. Hybridization capture approaches are known to suffer from off target capture and capture of highly repetitive sequences. Recently, a low cost PCR based method has been designed that utilizes multiplex ligation dependent probe amplification (MLPA probes) of enriched library followed by capillary electrophoresis to validate exome library in terms of enrichment efficiency to prevent sequencing of unsuccessfully enriched libraries (Klonowska et al., 2016).

## Exome Sequencing: An Edge Over Other Approaches

Whole exome sequencing (WES) holds certain advantages over its comparable approaches, like RNA sequencing, that is sometimes limited by the bias of transcript abundance and also dependent on tissue and stage while exome sequencing permits exploration of the genes and alleles. Similarly, sequencing of gene rich regions by methylation filtration method is limited by the minimum control a researcher can execute on specific target regions especially during genotyping of diverse germplasm in parallel. While exome analysis is a probe-based enrichment approach that targets specific regions rather than random euchromatic regions. Other methodologies viz., EST sequencing, high Cot DNA selection (Barbazuk et al., 2005) are less efficient in delivering specific sequences in targeted manner (Fu et al., 2010).

Exome sequencing serves a great advantage when WGS is either not practical or necessary and as an adjunct to later. It allows affordable dissection of subregions of complex genomes and reduction of non-pertinent repetitive sequences to confer several benefits over WGS: (a) multiplexing of more samples for a given sequencing space, (b) sequencing of targeted informative regions reduces the complexity of data analysis, (c) identification of functional molecular markers, (d) useful platform to collect genomic data at population level for evolutionary and phylogeny studies, (e) existing databases provides functional context for exome identified SNPs through transcript/exon annotation in contrast to SNPs identified outside coding regions through genotyping based sequencing which are not easily annotated (Scheben et al., 2017), and (f) provides high coverage for identification of low frequency sequence variants (Klonowska et al., 2016) for complex traits (Kiezun et al., 2012). Exome sequencing represents an established method of targeted resequencing of gene space (Bamshad et al., 2011) especially for phylogenetics and divergence studies by targeting evolutionary more conserved regions, i.e., specific genes or genomic regions and thus provides improved sequencing depth of targeted regions rather than by surveying the entire genome.

Though high-depth WGS is considered as a gold standard for sequencing and re-sequencing purposes as it can access and interrogate all regions of genomes, exome sequencing still forms an alternative method of choice for the trait or target specific studies. It has demonstrated its potential in generating genome wide data for species such as black cotton wood (Zhou and Holliday, 2012), pine (Neves et al., 2013), and sugarcane (Song et al., 2016) etc., which are intractable for WGS studies. Since hybridized captured fragments are longer than probes used to retrieve them, exome sequencing provides insights into coding, intronic, UTRs and putative regulatory regions for a comprehensive view of gene regulation.

## Exome Sequencing and Phylogenetic Studies

Phylogenetic studies rely on the information content of homologous genomic regions across diverse taxonomic groups. Due to general conservation of exons of protein coding sequences as well as their inherent variability in third codon position, probes designed from the same ensures the efficacy of target capture in reference species and also holds potential to extend their capture across diverse taxas to resolve topologies at moderate to deep evolutionary time scales. Introns are generally targeted to analyze phylogeny at or below the species level. Apart from probe design, loci under consideration, target number, size and its properties, population size and time scale under study are other important determinants to consider during phylogeny analysis using sequence capture.

Relative to other approaches viz. use of restriction enzymes, PCR amplification, genome skimming or WGS approaches, phylogenetic analysis using sequence capture via hybridization offers certain advantages, such as, (a) ability to enrich for degraded DNA samples obtained from museums or herbariums; (b) reduced sequencing of contaminant DNA from paleontologiocal samples; (c) targeted approach to reduce genome complexity relative to anonymous loci produced by restriction enzymes based approaches; (d) multiplexing offers broader sampling and deep coverage relative to WGS to target phylogenetically informative loci to resolve topologies with increased resolution in a cost effective manner without involving complexities of data analysis and storage at whole genome scale; and (e) deep coverage of targeted and unlinked loci allows to accommodate for more variation in probe designs to generate family wide sets of probes that ensures high resolution and strong bootstrap values for phylogenetic trees (de Sousa et al., 2014). These features form the forte for wide applicability of sequence capture tools through probe hybridization in phylogenetic studies (**Table 1**) (Jones and Good, 2016). Additionally, targeted enrichment of a sufficient number of informative loci prior to sequencing ensures phylogenetic accuracy to deduce broader and high resolution topologies. Whereas, selection of loci post-sequencing could cause systematic errors during analysis of the enormous amount of data generated at whole genome scale, that apart from targeted information also includes low quality, unusable data or data that cannot be effectively modeled. Since, the science of evolution and ecology is not dependent on sequencing of each base of the whole genome, rather it relies on deep sequencing of informative loci with desired evolutionary rates to produce well-resolved topologies.

**Table 1:** Crop species targeted for exome sequencing.

Features		Crop	Reference
Phylogenetic analysis		Hordeum	Brassac and Blattner, 2015
		Arecoideae	de Sousa et al., 2014
		Asteraceae	Mandel et al., 2014
		Milkweed	Weitemier et al., 2014
Genomic diversity		Maize	Fu et al., 2010; Liu et al., 2012; Muraya et al., 2015
		Wheat	Saintenac et al., 2011; Winfield et al., 2012
		Soybean	Haun et al., 2011
		Sugarcane	Song et al., 2016
		Barley	Mascher et al., 2013
		Brassica	Clarke et al., 2013
		Black Cottonwood	Zhou and Holliday, 2012
Monitoring alien introgression		Wheat	Winfield et al., 2016
		Barley	Wendler et al., 2014
Mutation characterization		Soybean	Bolon et al., 2011
		Rice	Henry et al., 2014
		Wheat	King et al., 2015; Krasileva et al., 2017
Mutagenesis + R gene enrichment		Wheat	Steuernagel et al., 2016
Trait mapping	Antioxidant metabolism	Tomato	Ruggieri et al., 2016
	Wood property traits	Eucalyptus	Dasgupta et al., 2015
	Flowering time regulatory gene	Brassica	Schiessl et al., 2014
	Many nodded dwarf phenotype	Barley	Mascher et al., 2014
	Early maturation	Barley	Pankin et al., 2014
	Biomass production	Maize	Muraya et al., 2015
Sequence based genetic mapping		Brassica	Galvão et al., 2012; Clarke et al., 2013
		Pine	Neves et al., 2014
		Cassava	Pootakham et al., 2014
		Wheat	Allen et al., 2013


Though, initially most of the targeted sequencing effort to define phylogenetic relations were directed toward plastome or ribosomal DNA sequences, recently, Mandel et al. (2014) utilized conserved orthologous set (COS) loci from the nuclear genome of Asteraceae (Chapman et al., 2007) to deduce the phylogenetic relationship across its 15 species and also demonstrated usability of 763 COS loci to facilitate taxonomic reconstruction in consistency with known phylogenetic relationships. Weitemier et al. (2014) further combined sequence capture with genome skimming and performed target enrichment for both low copy nuclear genes and genes from organelle genome to analyze phylogeny among 12 milkweed genotypes. The technique was named as Hyb-Seq and utilized enrichment probes designed from taxon specific genomic and transcriptomic data. Recently, Comer et al. (2015) compared the efficacy of hybrid gene capture and Long range PCR to describe phylogeny across 31 members of Arecoideae tribe and 5 outgroup taxas. de Sousa et al. (2014) utilized 50 low copy number (LCN) genes to describe Medicago phylogenetic. LCN genes are characterized by properties that are sufficiently informative for their use in phylogenetic analysis, such as high evolutionary rate, biparental inheritance (Small et al., 1998; Hughes et al., 2006), stable copy number, and provides higher resolution in comparison to ribosomal DNA or Plastome. Similarly, Brassac and Blattner (2015) analyzed 12 nuclear single copy loci and 1 chloroplast loci to infer phylogeny relations of *Hordeum* taxa and reported utility of multilocus species level phylogeny without sequencing whole genomes. Thus, the approach permits to target variation in much broader gene pool by accommodating a large number of genomes to be sequenced with less complexity in data analysis, in comparison to resequencing of whole genomes of same number of genotypes.

## Applications of Exome Sequencing for Crop Improvement

Apart from phylogenetic studies, WES also holds a wide applicability for crop improvement (**Table 1**) namely in (a) investigation of the molecular basis of natural variation and induced mutation, (b) identification of new genetic markers and rare variants, (c) and as an effective alternative for genomics studies of non-model and related wild species.

One of the first successful application of sequence capture was to enrich for 2.3 Mb chromosome interval and set of dispersed 43 genes in maize (Fu et al., 2010) and subsequently whole exome capture of maize to validate genetic diversity between cultivars (Liu et al., 2012) leading to identification of variation affecting biomass production (Muraya et al., 2015). Exome capture has been explored to identify natural variation and genetic diversity between wild and cultivated durum wheat accessions (Saintenac et al., 2011), hexaploid wheat varieties (Winfield et al., 2012), identification of novel co-dominant SNP markers (Allen et al., 2013) and monitoring of alien introgressions to ensure linkage drag reduction (Winfield et al., 2016). Exome capture in soybean confirmed the presence of unwanted intracultivar heterogeneity influencing observed variation in cv. Williams 82 (Haun et al., 2011). Barley exome was characterized to identify natural variation (Mascher et al., 2013), mutation involved in early maturation (Pankin et al., 2014), gene causative of many nodded dwarf phenotype (Mascher et al., 2014) and to differentiate between markers of *H. vulgare* and *H. bulbosum* for alien introgressions (Wendler et al., 2014). Exome sequencing has played a crucial role in accessing genomic variation of important perennials, like miscanthus^1^, black cotton wood (Zhou and Holliday, 2012) and pine (Neves et al., 2013) so as to better link their genotypes to phenotypes for agricultural improvement. Similarly, eucalyptus exome has been analyzed to identify mutation for wood property traits, wherein targeted resequencing for 94 genes functioning in different steps of secondary xylem formation served as an efficient strategy to identify SNPs and SNVs to develop mapping pedigrees (Dasgupta et al., 2015). Exome capture has been successfully utilized to generate high density gene rich genetic map in pine (Neves et al., 2013) and also for sequence based genetic mapping in brassicacea (Galvão et al., 2012). Thus, it is a highly relevant approach to examine genome synteny, identification of QTLs and candidate regulatory genes as well as to assist in genome assembly of species without reference genome. Sequence variation of 378 genes and associated regulatory regions involved in antioxidant metabolism was analyzed for 44 tomato landraces identified 4000 rare variants with >40X coverage (Ruggieri et al., 2016), thus demonstrating the capacity of exome sequencing to extend the knowledge of the genetic base (Gasc et al., 2016). Similarly, Pootakham et al. (2014) utilized target capture to genotype 100 F1 progeny of cassava mapping population segregating for starch viscosity phenotypes. Clarke et al. (2013) performed exome capture to target variation in 47 genomic regions of 10 brassica genotypes and characterized 589367 SNPs in QTL regions associated with nutritional or agronomically important traits such as yield, seedling vigor, seed quality, blackleg disease resistance, etc. Efficacy of sequence capture to analyze variation between homologs for flowering time regulatory gene in *B. napus* (Schiessl et al., 2014) open avenues for its usage to decode other important pathways in polyploid crops.

Cross capturing of exomes using probes of related species have been effectively applied for less resourced species. Extensive collinearity shared between sorghum and sugarcane genic regions (Wang et al., 2010) was exploited to enrich 5.8 Mb region of two sugarcane genotypes (Bundock et al., 2012). Till date, no reference genome sequence is available for sugarcane considering its genome structure (Zhang et al., 2012), but lately exome of 12 different accessions of *Saccharum* complex has been captured successfully (Song et al., 2016) to characterize natural allelic variation and haplotype pattern of 406 candidate genes, a first report on haplotype identification in polyploid sugarcane genome.

Suitability of exome capture to identify induced variants through mutagenesis to investigate gene function has been successfully demonstrated in many crops. Exome resequencing of four selected soybean mutants arose through fast neutron radiation confirmed presence of deletions (Bolon et al., 2011). Exon capture of mutagenized lines of rice (Henry et al., 2014) and wheat (King et al., 2015) has been done to identify novel mutations. MutRenSeq, i.e., exome capture of EMS lines in combination with RenSeq (R gene enrichment sequencing) was performed in hexaploid wheat for cloning Sr22 and Sr25 stem rust resistance genes (Steuernagel et al., 2016). More than 10 million mutations were cataloged in exome of wheat EMS lines and recessive mutation at vrn2 locus was identified in association with spring growth habit (Krasileva et al., 2017).

## Conclusion and Future Prospects

Exome sequencing via hybridization, holds high potential for its applicability in phylogenetic studies and genomic studies that require resequencing of multiple individuals of species with large and complex genome sizes. Few considerations have been raised for WES in terms of benefits conferred by WGS approaches, i.e., more consistent genome coverage, uniform distribution of sequencing quality parameters, identification of a large number of variants and absence of reference sequence bias generated by probes usage. But even in the near future, when prices of WGS will become at par with that of sequence capture via hybridization, the latter approach is much anticipated to sustain given its practical utilities for solving specific biological queries that rely on target sequencing. Resequencing of genomes of individuals in a mapping population/multiple accessions or cultivars of plants with large and complex genomes cannot be routinely employed and poses technical challenges in handling of robust informatics data. To its rescue, sequencing of sub-genomic regions is an effective alternate in terms of cost and efforts to achieve an equivalent depth of coverage, particularly for studies that do not require access to whole genomes.

## Author Contributions

PK and KG have made a substantial, direct and intellectual contribution to the work, and approved it for publication.

## Conflict of Interest Statement

The authors declare that the research was conducted in the absence of any commercial or financial relationships that could be construed as a potential conflict of interest.

## Footnotes

1 https://pag.confex.com/pag/xxi/webprogram/Paper5474.html
